# Effect of Different Cooking Methods on the Aroma and Taste of Chicken Broth

**DOI:** 10.3390/molecules29071532

**Published:** 2024-03-29

**Authors:** Can Yuan, Chengjian Xu, Lilan Chen, Jun Yang, Mingfeng Qiao, Zhoulin Wu

**Affiliations:** 1 College of Food, Sichuan Tourism University, Chengdu 610100, China; 2 Cuisine Science Key Laboratory of Sichuan Province, Sichuan Tourism University, Chengdu 610100, China; 3 Meat Processing Key Laboratory of Sichuan Province, Chengdu University, Chengdu 610106, China

**Keywords:** chicken soup, cooking methods, combi oven, aroma and taste, amino acids, key aroma compounds

## Abstract

A single combi oven, known for its versatility, is an excellent choice for a variety of chicken soup preparations. However, the impact of universal steam ovens on the flavor quality of chicken soup remains unclear. This study aimed to explore the impact of different cooking methods on the aroma and taste of chicken soup. Three cooking methods with various stewing times were compared: ceramic pot (CP), electric pressure cooker (EPC), and combi oven (CO). Analyses were conducted using electron-nose, electron-tongue, gas chromatography–ion mobility spectrometry (GC–IMS), automatic amino acid analysis, and chemometric methods. A total of 14 amino acids, including significant umami contributors, were identified. The taste components of CP and CO chicken soups were relatively similar. In total, 39 volatile aroma compounds, predominantly aldehydes, ketones, and alcohols, were identified. Aldehydes were the most abundant compounds, and 23 key aroma compounds were identified. Pearson’s correlation analyses revealed distinct correlations between various amino acids (e.g., glutamic acid and serine) and specific volatile compounds. The aroma compounds from the CP and CO samples showed similarities. The results of this study provide a reference for the application of one-touch cooking of chicken soup in versatile steam ovens.

## 1. Introduction

Chicken meat is a valuable source of essential nutrients, including vitamins, amino acids, and collagen, for the human body. Phospholipid nutrients present in chicken play a vital role in human growth and development, making them significant contributors to the fat and phospholipid intake in the Chinese diet [1]. Fresh chicken meat has a subtle rawness, metallic taste, and hint of saltiness. Stewing chicken is a widely adopted cooking technique in China [2]. Simmering chicken in soup enhances its flavor by releasing high-quality proteins, creatinine, functional peptides, umami peptides, and free amino acids [3]. This enhances the flavor and fragrance of chicken soup, rendering it more easily digestible and absorbable [4,5]. In Sichuan cuisine, chicken soup, whether clear or milk-based, is commonly used as an additive to dishes, greatly enhancing their umami taste and gaining popularity among consumers [5,6].

The flavor of chicken soup is a key determinant of its quality and desirability, with aroma and taste being the crucial components [7]. Existing research has predominantly explored the effects of cooking time, temperature, and salt addition methods on the aroma and taste of chicken soup [4,8,9,10]. As crucial taste components and aroma precursors, free amino acids contribute to diverse taste sensations, including sweetness, acidity, and bitterness [11]. For instance, serine, alanine, glycine, threonine, lysine, proline, and hydroxyproline impart sweetness, while aspartic acid and glutamic acid contribute to acidity and serve as important precursors for umami substances [12]. Arginine, histidine, methionine, valine, leucine, isoleucine, phenylalanine, tyrosine, and tryptophan produce bitterness. The Maillard reaction, involving the interaction of free amino acids with reducing sugars, significantly contributes to the development of meat flavors. From the Maillard reaction, key aroma compounds identified in chicken broth include 3-methylthiopropanal, 2-methyl-3-mercaptofuran, methylpyrazine, 2-ethyl-4-methylthiazole, and (*E*, *E*)-2,4-nonadiena [13,14,15]. The identification of aroma compounds in chicken broth has been conducted using gas chromatography (GC), GC–ion mobility spectrometry (GC–IMS), GC–mass spectrometry (GC–MS), and GC–olfactory determination (GC–O) [3,5,10,16]. In particular, GC–IMS has recently emerged as a cutting-edge technology for gas-phase separation and detection. In comparison to GC–MS, GC–IMS offers the advantages of user-friendly operation, robust separation capabilities, shorter analysis times, heightened sensitivity, and preservation of the sample’s original flavor. GC–IMS analytical technology has broad applications for analyzing volatile flavor compounds and conducting quality testing for diverse food products [17,18]. However, its application in the study of volatile aroma substances in chicken broth is relatively limited.

The rise of the social economy and acceleration of urbanization have generated an increased demand for kitchen processing equipment within the catering industry. To cater to the evolving needs of the catering industry, this study concentrated on how stewing technology influences the quality of chicken soup, aiming to offer consumers a more user-friendly cooking experience [1,8]. Lai et al. [4] explored the impact of different cooking modes (utilizing stainless-steel pots, ceramic pots, and electric ceramic stewpots) on the chemical compositions, whiteness, 5′-nucleotides, fatty acids, sensory quality, and flavor substances in chicken soup. Additionally, Zou et al. [19] investigated the chemical composition and sensory characteristics of pork rib and Silkie chicken soups and examined the effects of various cooking techniques.

In recent years, combi ovens have emerged as highly versatile and advanced kitchen appliances with applications in both professional and home kitchens. This innovative cooking technique combines various methods, including convective cooking, steam cooking, combination cooking, and stewing, into a single unit. Notably, the combi oven excels in steaming vegetables, fish, and other foods as well as in baking and roasting dishes, making it an ideal choice for a diverse array of culinary preparations. Combi ovens have been widely adopted in commercial kitchens, such as restaurants, bakeries, and catering services, owing to their flexibility and efficiency [20,21,22]. While existing research on combi ovens primarily explores the production process of dishes, such as whole fish frying, sweet and sour spare ribs, and barley meat, there is a noticeable gap in understanding the impact of universal steam ovens on the flavor quality of chicken soup.

To address this gap, the aim of this study was to examine how different cooking methods (ceramic pot [CP], electric pressure cooker [EPC], and combi oven [CO] with varying stewing times) influence the aroma and taste of chicken soup. This investigation employed advanced tools such as electron-nose (E-nose), electron-tongue (E-tongue), GC–IMS, automatic amino acid analysis, and chemometric methods. The results of this study lay the foundation for optimizing chicken soup production using smart kitchen equipment.

## 2. Results and Discussion

### 2.1. E-Nose Analysis

**Figure 1:**
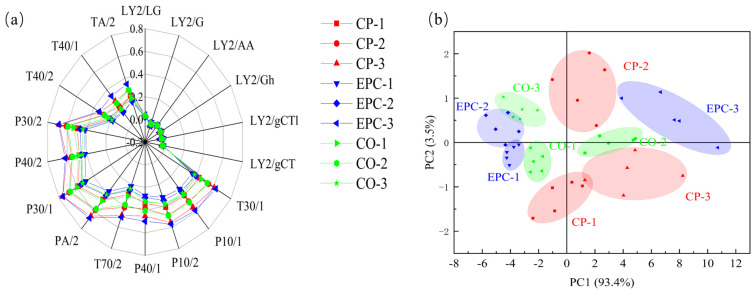
Electronic nose (E-nose) radar plot of different chicken soup samples. CP, ceramic pot; EPC, electric pressure cooker; CO, combi oven. (**a**) Radar map of the E-nose; (**b**) PCA of the E-nose.

An E-nose was used to detect and differentiate complex aroma substances in chicken soup. E-nose sensors with low odor thresholds enable the identification of chemical variation during chicken soup stir-frying. A radar chart was used to show the differences in the average sensor array responses to the samples (Figure 1). The chicken soup samples exhibited high response values from 12 sensors (P40/1, P40/2, P10/1, P10/2, T40/1, T40/2, T70/2, T30/1, P30/1, P30/2, PA/2, and TA/2), indicating the generation of alcohols, fluorides, chloride combustion products, aldehydes, hydrogen sulfide, ammonia, amine compounds, and aromatic compounds during cooking. These 12 sensors effectively distinguished the chicken soup samples based on different cooking times and methods. In the CP and EPC methods, the response values increased with extended cooking time, whereas, in the CO method, the response value peaked at a stewing time of 2 h. However, chicken soup samples showed no response to six sensors (LY2/LG, LY2/G, LY2/AA, LY2/Gh, LY2/CT1, and LY2/GCT), suggesting the generation of sulfides, nitrogen oxides, butane, propane, and acetone during cooking. The results indicated minimal differences among these substances, whereas the remaining sensors exhibited significant differences in nonpolar substances (hydrocarbons, ammonia, and chlorine), polar substances, aromatic substances (toluene and xylene), chlorine, amines, and other substances, suggesting substantial variations in these types of substances among the samples.

PCA was conducted to analyze multivariate data while considering variable quantity restrictions [23]. The aromatic components in chicken soup samples processed using different cooking methods were further studied using PCA as a non-targeted statistical method (Figure 1). As shown in Figure 1b, the cumulative contribution rates of principal components 1 (93.4%) and 2 (3.5%) reached 96.9%, providing a comprehensive reflection of the overall sample information. Consequently, the coordinates primarily represented by principal component (PC1) captured the odor characteristics of the samples. The dispersion of data points for the chicken soup samples indicated substantial diversity in their odor characteristics. The aroma components in the chicken soup exhibited little overlap, underscoring the effectiveness of PCA in distinguishing between the various samples. The contribution ratio of PC1 significantly outweighed that of PC2. The greater the horizontal distance of the samples in PC1, the more pronounced the olfactory differences between them. The horizontal coordinate distance distribution indicated similarities in the aroma of CP-2, CO-2, CO-1, CO-3, and EPC-3; conversely, the distance distributions of the other samples (EPC-1, EPC-2, CP-1, and CP-3) were noticeably further apart. This suggests an evident difference in aroma between the latter sets of samples.

### 2.2. E-Tongue Analysis

**Figure 2:**
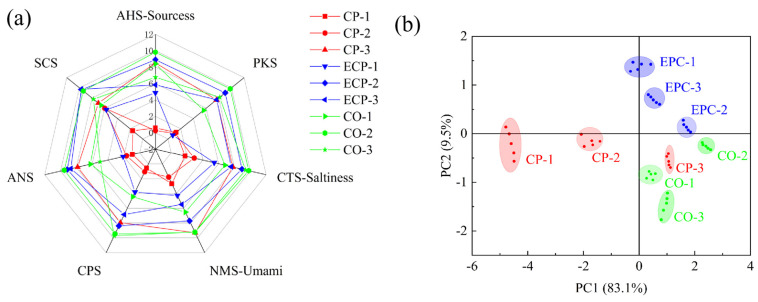
Electronic tongue (E-tongue) radar plot of principal component analysis (PCA) results and a two-dimensional image of the E-tongue date for different chicken soup samples. CP, ceramic pot; EPC, electric pressure cooker; CO, combi oven. (**a**) Radar map of the E-tongue; (**b**) PCA of the E-tongue.

To detect and distinguish the complex taste characteristics of chicken soup, an E-tongue was employed [24]. In contrast, the E-tongue emulates the taste organs of the human oral cavity using an intelligent biomimetic system, enabling the detection of sample flavors [12]. Unlike conventional sensory evaluations, the E-tongue mitigates the subjective influence of human consciousness on sensory assessments and diminishes the impact of external factors. Figure 2a shows a radar chart illustrating the response values of the E-tongue sensors, revealing the differences in taste characteristics among the chicken soup samples. Noticeable variations were observed in the relative intensity values of the E-tongue sensors, demonstrating their effectiveness in distinguishing taste differences among different samples [25]. For instance, EPC-2 recorded the maximum response levels on the AHS-sources, CTS-saltiness, PKS, ANS, and NMS-umami sensors. Conversely, CP-3 exhibited the highest response among the SCS sensors. The heightened response of EPC-2 to sourness, saltiness, and umami taste was attributed to the elevated concentration of salt-contributing substances (including ionized organic and inorganic matter) in its aqueous solution. Moreover, sample EPC-2 contained a substantial concentration of umami-contributing substances, such as amino acids and nucleotides.

As shown in Figure 2b, PC1 and PC2 explained 83.1% and 9.5% of the total variation in chicken soup samples, respectively, with PC1 making a more substantial contribution. PC1 and PC2, with a cumulative contribution rate of 92.6%, effectively captured and reflected the overall flavor profiles of the nine chicken soup samples. Sample data points showed concentrated patterns, indicating the stability of the E-tongue results for chicken soup. Distinct distributions across the four quadrants were observed for the data points from the various chicken soup samples, indicating noticeable taste differences. The weight of PC1 was more significant than that of PC2. Consequently, even samples closely positioned along PC1 exhibited substantial taste differences [26]. As shown in Figure 2b, EPC-3 and CO-1 occupied the first and fourth quadrants, respectively. The primary distinction was attributed to PC2, which indicated a high degree of similarity. Similarly, CP-3 and CO-3, located in the fourth quadrant, exhibited relatively similar horizontal coordinate distribution distances, suggesting a high degree of similarity. In contrast, noticeable differences were observed between CO-2, EPC-2, EPC-1, CP-2, and CP-1.

### 2.3. FAA Analysis

FAAs are crucial flavor substances that are closely linked to taste formation, enabling the differentiation of tastes among various samples (Table 1). A total of 14 amino acids were identified in the chicken soup, with total free amino acids (TFAAs) ranging from 28.17 to 42.75 mg/100 g. Categorized by taste intensity, these amino acids fall into umami (Asp, Glu), sweet (Gly, Ala, Thr, Ser), and bitter (Val, Ile, Leu, Phe, His, Tyr, Lys, Arg) groups [27]. The order of amino acid content based on different flavor types is as follows: CP-3 > CO-3 > CP-2 > CO-2 > CP-1 > CO-1 > EPC-3 > EPC-1 > EPC-2 for umami; CP-3 > CP-2 > CO-3 > CP-1 > CO-2 > EPC-1 > CO-1 > EPC-2 > EPC-3 for sweet; and CP-2 > CP-3 > CO-3 > EPC-2 > EPC-3 > CP-1 > CO-1 > CO-2 > EPC-1 for bitter. It is noteworthy that the amino acid umami content sequence differed from the relative strength values observed with the E-tongue, possibly because of the diverse taste nucleotide contents in different samples [28].

The TAV serves as an indicator for assessing the contribution of flavor substances to the overall taste of a sample. TAV is calculated by dividing the concentration of a specific taste-active compound in a food sample by its taste threshold. TAV > 1 signifies a substantial impact on taste characteristics, whereas a TAV < 1 suggests a modifying influence on the sample’s taste [29]. Table 1 presents the amino acid TAV of the chicken soup samples. Notably, only the TAV value of glutamic acid exceeded 1, indicating that umami amino acids significantly influenced the overall taste of all samples. In contrast, the TAV values for sweet amino acids in all samples were <1, suggesting a modifying role in the overall taste. Additionally, the TAV values for bitter amino acids were <1, indicating that their taste activity was typically masked by umami and sweet flavors. These results highlight the significant contribution of amino acids, especially in Sample CP-3, to the overall taste.

**Figure 3:**
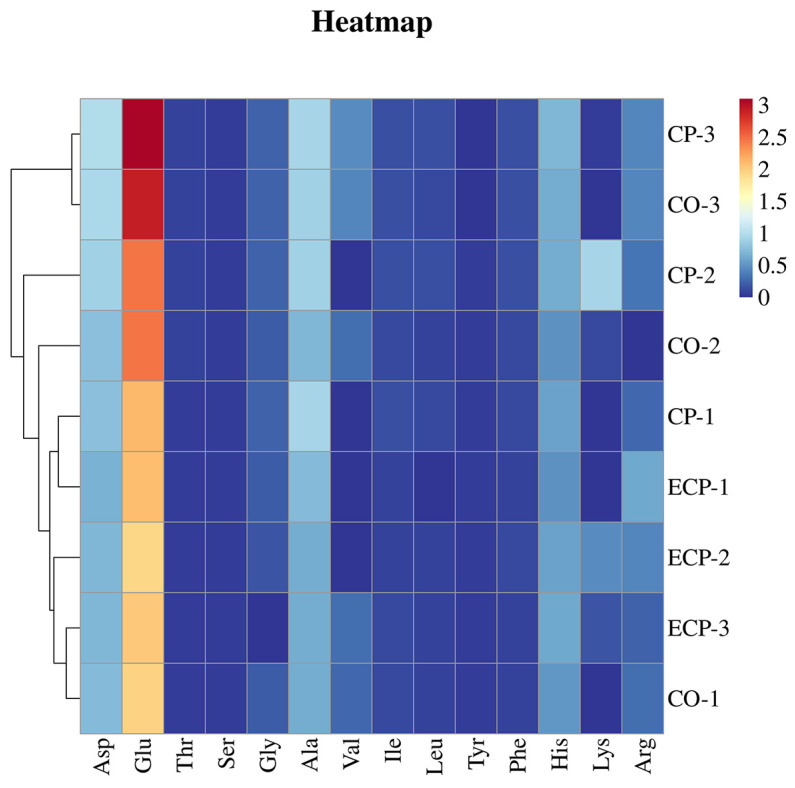
Cluster analysis of free amino acids in chicken soup samples. CP, ceramic pot; EPC, electric pressure cooker; CO, combi oven.

Subsequent analysis of FAAs in the chicken soup resulted in another heat map based on TAV, as shown in Figure 3. The results reveal significant variation in the FAA compositions of the chicken soups. Notably, CP-3 and CO-3 clustered together, whereas CP-1, EPC-1, EPC-2, EPC-3, CO-1, and CO-2 formed separate groups. CP-2 was distinctly positioned within its own cluster. In summary, TAV analysis showed that the taste components of amino acids in chicken soup prepared using CP and CO were relatively similar.

### 2.4. GC–IMS Analysis

**Figure 4:**
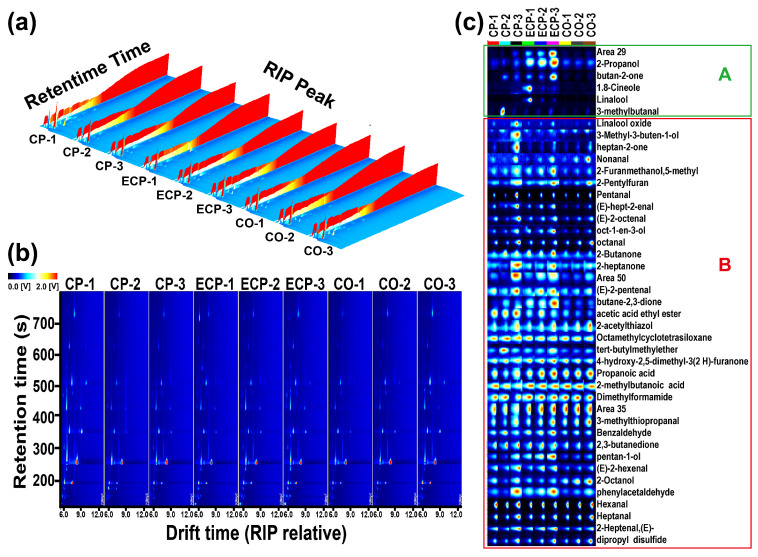
Gas chromatography–ion mobility spectrometry (GC–IMS) topographic and fingerprint chromatogram volatile compounds in samples of Pixian broad bean paste. (**a**) Three-dimensional topographic image; (**b**) Two-dimensional spectrum; (**c**) Fingerprint chromatogram volatile compounds. CP, ceramic pot; EPC electric pressure cooker; CO, combi oven; Region A, the aroma compounds exclusive to chicken soup prepared by a single method; Region B, almost all volatile aroma compounds present in all samples.

The GC–IMS spectra of the chicken soup prepared using the three distinct methods were visually examined to identify potential distinctive signals [12]. Figure 4a shows the three-dimensional spectra of volatile aroma compounds in chicken soup during the cooking process, where the vertical axis denotes the retention time in GC separation and the horizontal axis represents the ion migration time in IMS separation. Each peak in the reaction ion peak (RIP) signifies a volatile aromatic substance [30]. Notably, the concentrations of aromatic compounds varied with cooking method and duration [18]. Comprehensive analysis of the GC–IMS profiles is shown in Figure 4b. In this two-dimensional plot, each point flanking the reaction ion peak (RIP) signifies a distinct volatile organic compound. The color intensity of the points corresponds to the area size, indicating higher concentrations (red hues represent high intensity, blue hues represent low intensity). Some compounds exhibited multiple spots, denoting different dimers or trimers with distinct properties and concentrations. Figure 4b shows no generation or disappearance of RIP points; instead, there is variation in the color intensity of RIP points. The analysis suggested minimal divergence in the GC–IMS spectra among the different chicken soups. Variations in aroma substances within each chicken soup sample primarily involved the quantity, position, time, and intensity of the ion peaks. Such differences may arise from factors such as water evaporation during cooking, nutrient dissolution, and the oxidative decomposition of certain nutrients.

This study quantified aromatic compound levels in diverse chicken soups and established their characteristic flavor fingerprint profiles. The brightness of each compound’s signal corresponds to its intensity, with brighter signals indicating stronger intensity and darker signals indicating weaker intensity [17]. In Figure 4c, rows represent chicken soup prepared by three different methods and columns signify a specific aromatic compound across various samples (darker red signals indicate a relatively higher content of that substance). On the fingerprint profile, two entities with the same name represent the monomeric and dimeric forms of the compound. In Region A, aroma compounds were exclusively present in chicken soup prepared using a single cooking method. Aroma compounds such as 2-propanol, butun-2-one, 1, 8-cineole, and linalool were present in chicken soup prepared using an electric pressure cooker, while 3-methybutanal was detected in CO-2. In contrast to the aroma compounds exclusive to chicken soup prepared by a single method in Region A, all aroma compounds in Region B were detected in chicken soup prepared through all three cooking techniques. In Region B, almost all volatile aroma compounds, such as aldehydes, ketones, and alcohol, were present in all samples, with concentrations rising as stewing time increased. The content of almost all volatile aroma compounds increased with stewing time.

We also performed a qualitative examination of volatile flavor compounds during chicken soup simmering using the GC–IMS built-in NIST and IMS databases. The identification process considered the retention indices, retention times, and migration times (Table S1). GC–IMS analysis identified 39 volatile aroma compounds, comprising 14 aldehydes, seven ketones, nine alcohols, two acids, two heterocyclic compounds, two ethers, and three others.

**Figure 5:**
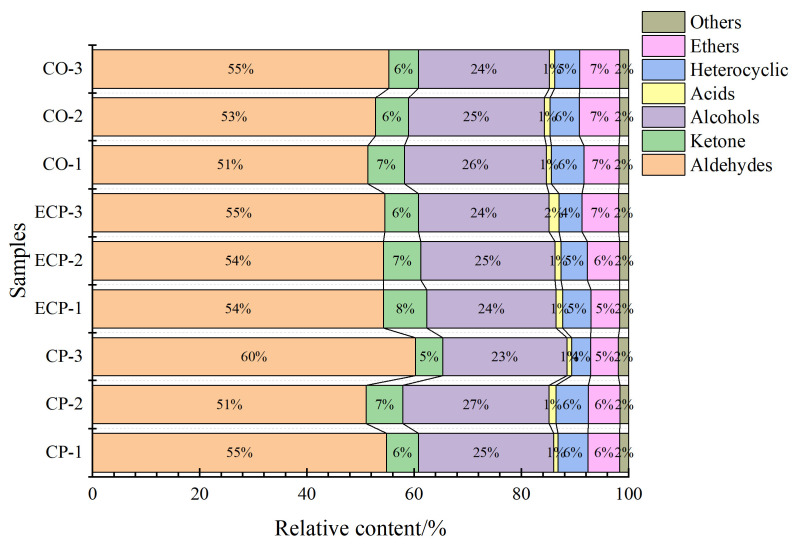
Relative contents of different compounds in chicken soup. CP, ceramic pot; EPC electric pressure cooker; CO, combi oven.

As shown in Figure 5, aldehydes exhibited the highest content in each sample, ranging from 51% to 60%, with no pronounced differences among the samples. Aldehydes primarily result from the oxidative breakdown of fats, feature a low threshold, and serve as vital volatile flavor compounds in chicken broth. These compounds can further react with other substances, generating diverse flavor profiles [4,19]. The samples contained various aldehydes, including (E)-2-octenal, (E)-2-heptenal, (E)-hept-2-enal, octanal, heptanal, hexanal, phenylacetaldehyde, (E)-2-hexenal, benzaldehyde, 3-methylthiopropanal, pentanal, (E)-2-pentenal, nonanal, and 3-methylbutanal. (E)-2-octenal imparts a citrusy aroma with fruity undertones, whereas octanal imparts a citrus-like scent with orange nuances [19]. (E)-2-pentenal adds a fruity note, (E)-heptenal provides a grassy fragrance with green undertones, and (E)-hept-2-enal is associated with a green leafy scent [4]. (E)-2-hexenal is recognized for its green and leafy aroma, whereas phenylacetaldehyde contributes a sweet, floral fragrance. Nonanal is characterized by a floral and waxy aroma, while benzaldehyde has an almond-like scent, 3-methylthiopropanal adds a pungent, sulfuric note, and 3-methylbutanal contributes a malty fragrance to chocolate undertones. Heptanal adds a fruity note with a waxy undertone and (E)-2-pentenal imparts a green and fatty character to the aroma. Nonanal features a floral, waxy aroma, whereas pentanal contributes to a pungent, fruity odor [4,31].

The ketone content in chicken broth ranged from 5% to 8%, with minimal variability observed between samples. These compounds significantly contribute to the flavor profile of fermented meat products, deriving mainly from *β*-keto acid decarboxylation, incomplete *β*-oxidation of free fatty acids, and amino acid substance degradation [4,9,19]. The 2,3-butanedione imparts a buttery and creamy aroma. Heptan-2-one contributes a sweet and fruity fragrance. Butan-2-one adds a slightly sweet and solvent-like aroma, 2-heptanone is characterized by a fruity and floral scent, and 2-butanone, also known as methyl ethyl ketone, has a sweet and acetone-like odor. The 4-hydroxy-2,5-dimethyl-3(2H)-furanone imparts a sweet, caramel-like aroma with fruity undertones, while butane-2,3-dione may contribute to the overall aroma owing to its characteristic scent, including a buttery fragrance, a fermented scent, and a creamy aroma [31].

Alcohols, which are renowned for their association with plant and floral aromas, play a pivotal role in enhancing food flavor. In fermented chicken broth, these alcoholic compounds primarily originate from lipid degradation [31]. Unsaturated fatty alcohols with lower thresholds can potentially enhance overall flavor. However, alcohols with higher flavor thresholds exert a comparatively minor impact on taste [32], constituting 21–28% of the total composition. Noteworthy examples include oct-1-en-3-ol, which imparts a woody, citrus-like aroma, and 2-octanol, imparting a mild, sweet, and alcoholic scent. Pentan-1-ol adds a slightly fruity and floral note, while 2-propanol (isopropanol) exhibits the characteristic alcohol odor. Other contributors include 3-methyl-3-buten-1-ol with a fruity and floral aroma, and linalool oxide, which contributes to sweet and floral fragrance. Additionally, 2-furanmethanol, 5-methyl-, adds a sweet and caramel-like note, and 1,8-cineole (eucalyptol) imparts a fresh and camphoraceous aroma [10]. Alcohol content within this range significantly influenced the sensory characteristics of the chicken broth.

However, acids, heterocyclic compounds, ethers, and others, when present at relatively low concentrations, make a noteworthy contribution to the overall flavor [16]. For example, propanoic acid imparts a sour and slightly rancid aroma. Similarly, 2-methylbutanoic acid adds a cheesy and sweaty scent, 2-pentylfuran contributes a sweet and caramel-like note, and 2-acetylthiazol imparts a roasted and nutty fragrance [33]. Dipropyl disulfide adds a pungent and garlic-like aroma, whereas tert-butylmethylether has a faintly sweet and ether-like odor [34]. Acetic acid ethyl ester contributes to the fruity and sweet aroma, whereas dimethylformamide is characterized by a faint, amine-like odor [35]. Despite their relatively low amounts, these substances exert a discernible influence on the overall flavor of chicken broth.

### 2.5. Key Volatile Aroma Compounds

**Figure 6:**
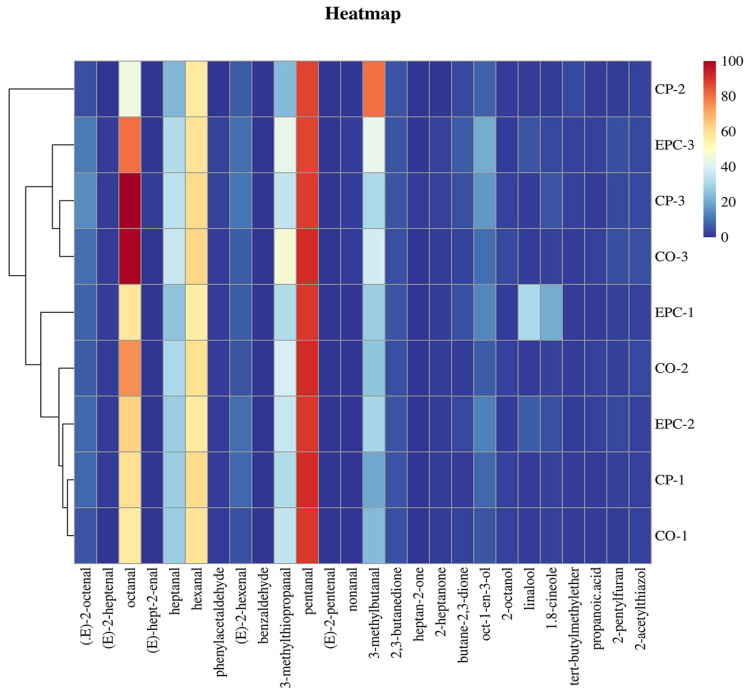
Cluster analysis of key volatile aroma components with relative odds activity values (ROAV) of >1 in chicken soup. CP, ceramic pot; EPC, electric pressure cooker; CO, combi oven.

The influence of key volatile aroma compounds on the overall aroma profile was determined not only by their individual concentrations but also by their ROAV values. Compounds with higher ROAV contribute more significantly to overall aroma [30]. The volatile aroma compounds identified as key aroma compounds (ROAV ≥ 1) in chicken broth were visualized in a heatmap (Figure 6). The results show noticeable differences in the key volatile aroma compounds during the cooking process. The 23 identified key aromatic compounds were (E)-2-octenal, octanal, (E)-hept-2-enal, heptanal, hexanal, pentanal, phenylacetaldehyde, (E)-2-hexenal, 3-methylthiopropanal, nonanal, 3-methylbutanal, butane-2,3-dione, 2,3-butanedione, heptan-2-one, oct-1-en-3-ol, 2-octanol, linalool, 1.8-cineole, 2-heptanone, tert-butylmethylether, propanoic acid, 2-pentylfuran, and 2-acetylthiazol. Octanal had the highest ROVA of 100, making a substantial contribution to the overall flavor, with a citrus-like scent enriched with orange nuances [31]. Simultaneously, heptanal, hexanal, pentanal, 3-methylthiopropanal, and 3-methylbutanal, each with ROAV ≥ 30, significantly influenced the overall flavor profile. Heptanal imparts a fruity and waxy aroma, hexanal contributes a green and grassy fragrance, pentanal adds a pungent and fruity note, and 3-methylthiopropanal provides a pungent and sulfuric aroma [4,36]. Additionally, 3-methylbutanal imparts a malty and photoplate-like scent. This suggests that aldehydes play a pivotal role in shaping the impact of aromatic compounds on the overall flavor profile.

As shown in Figure 6, where blue corresponds to low content and red corresponds to high content, CP-3, EPC-3, and CO-3 were grouped together and showed no significant differences in the distribution of the key volatile components. CP-1, CO-1, CO-2, EPC-1, and EPC-2 samples shared a group of volatile aroma markers. CP-2 stood out as a separate category, indicating that the aromatic compounds in the CP and CO samples were relatively similar.

### 2.6. Correlation Analysis

**Figure 7:**
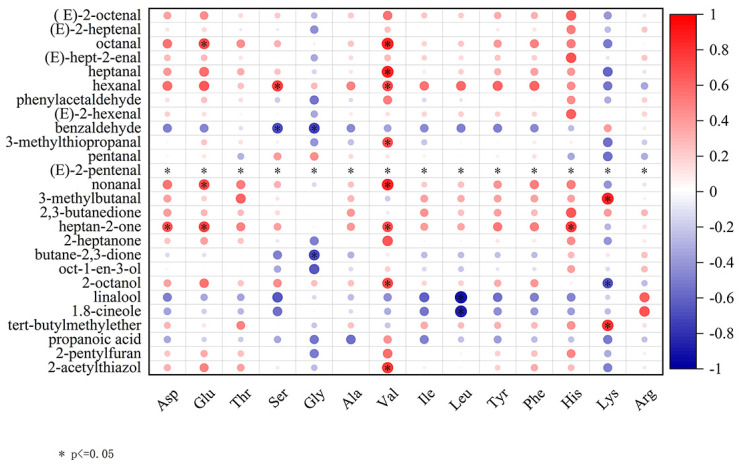
Correlation analysis between key volatile aroma components and free amino acids (FAA) during the stewing process by Pearson’s correlation analysis. The symbols “*” signify significant (*p* < 0.05) correlations, respectively. Positive (0 < r < 1) and negative (−1 < r < 0) correlations are displayed in red and blue, respectively.

Pearson correlation analyses were conducted to scrutinize the association between key volatile aroma compounds (ROAV ≥ 1) and FAAs. FAAs play a pivotal role as flavor precursors in foods, with aromatic volatile compounds responsible for flavor originating primarily from the Maillard reaction between FAAs and reducing sugars [3]. The decarboxylation and deamination of amino acids result in the formation of diverse alcohols and aldehydes [31]. Correlation analysis, as illustrated in Figure 7, revealed that each key volatile aroma compound was correlated with FAA. Glutamic acid (Glu) was positively correlated with octanal, non-anal, and heptan-2-one. Serine (Ser) displayed a notable positive correlation with hexanal and an inverse correlation with benzaldehyde. Glycine (Gly) was negatively correlated with benzaldehyde content. Glutamic acid and glycine are known to react with sugar compounds to produce flavor substances in meat [35]. Valine (Val) exhibited notable positive correlations with octanal, hexanal, heptanal, 3-methylthiopropanal, heptan-2-one, 2-octanol, and 2-acetylthiazol. Valine is a precursor of isobutyraldehyde, which is formed via the Maillard reaction [36]. Histidine (His) positively correlated with heptan-2-one, while lysine (Lys) positively correlated with 3-methylbutanal and tert-butylmethylether. Lysine can undergo the Maillard reaction to generate pyrazine compounds, imparting a meaty aroma to meat products [14]. Leucine (Leu) was negatively correlated with 1,8-cineole and linalool levels. Some reports have indicated that leucine undergoes a Maillard reaction with reducing sugars to form isovaleraldehyde [36]. (E)-2-pentenal was positively correlated with all amino acids.

This study has some limitations. First, we focused on specific cooking methods and their impact on chicken soup flavor, neglecting other potential factors influencing taste. Additionally, correlations do not necessarily establish causation, and further research is needed to explore the broader context of these associations. The study’s scope is confined to certain amino acids and volatile compounds, potentially missing other contributors to flavor complexity.

## 3. Materials and Methods

### 3.1. Materials

Sanhuang chicken (hens fed a commercial diet for 150 days), ginger, scallions, and salt were purchased from a local Yonghui supermarket (Chengdu, China). Hydrochloric acid (HCl; concentration ≥ 35%), phenol, and sulfosalicylic acid (analytically pure AR) were purchased from Chengdu Cologne Chemical Co., Ltd. (Chengdu, China).

### 3.2. Instruments and Equipment

The following instruments and equipment were used: a J2-MI High-speed centrifuge (Beckmen corporation, Brea, CA, USA); Fox 4000 electronic nose system and α-Astree electronic tongue system (Toulouse Alpha MOS, Toulouse, France); S-433D Fully Automatic Amino Acid Analyzer (SHYKAM LTD, Thedinghausen, Germany); FlavourSpec^®^ GC–IMS (Aerospace Center, Cologne, Germany); Electric pressure cooker (SY-50YC9086 Supor Co. Ltd., Hangzhou, China); and 20-2/1 iCombi Pro combi oven (Rational AG Co., Landsberg am Lech, Germany). 

### 3.3. Cooking of Chicken Soup and Sample Preparation

The chickens were cleaned, gutted, and washed with water before being weighed. Then, 30 chickens were halved, discarding the head, neck, claws, and visible fat, and uniformly chopped into pieces of approximately 3 ± 0.2 cm before being thoroughly mixed. The chicken soup was made with a carcass−ginger−water weight ratio of 100:2:300. Next, salt was added to the soup at a carcass−salt weight ratio of 100:1 approximately 10 min before the end of cooking. Nine chicken soups were produced using three different pots and stewing modes. Based on the literature and pre-experiments, the process parameters for preparing chicken soup using the casserole stew mode were obtained. The chicken broth ingredients and Combi oven were heated to 97 °C for 10 min (Ceramic Pot, CP) and 20 min (Combi oven, CO), and the temperature was maintained at 97 ± 1 °C. The stew mode was directly selected for the electric stew pot. During the cooking process, its power remained steady at 900 watts, while its pressure was sustained at 70 kPa, resulting in the pot’s temperature reaching its maximum value within 15 min. The stew mode was promptly selected for the universal steam oven, with a steam power output of 36 kW for optimal heating efficiency. The total stewing time for each type of chicken soup is presented in Table 2. After filtering the chicken soup through four layers of cotton gauze to eliminate solid residues, the clarified broth was stored at a temperature of 4 °C for further examination.

### 3.4. Physicochemical Measurements

#### 3.4.1. E-Nose Analysis

Chicken soup samples were analyzed using a FOX-4000 E-nose system (Alpha MOS, Nantes, France) with 18 metal oxide semiconductor sensors. A 2.0 g chicken soup sample was placed in a 100-mL vial and heated to 70 °C, and its headspace gas was injected into the sensor array at 2000 μL/s for a 300 s run. The sensors encompassed a diverse array of functionalities, with their order being rearranged as follows: LY2/LG (oxynitride and sulfides), LY2/G (ammonia, amine compounds, and oxynitride), LY2/AA (ammonia, alcohol, and acetone), LY2/Gh (ammonia and amine compounds), LY2/gCT1 (hydrogen sulfide), LY2/gCT (propane and butane), T30/1 (polar organic compound, hydrogen sulfide), P10/2 (methane and ethane), P10/1 (carbon-oxygen compound, ammonia, and chlorine), P40/1 (chloride and fluoride), T70/2 (aromatic compounds), PA/2 (alcohol, ammonia, and amine compounds), P30/1 (hydrogen sulfide, chlorine, and fluoride), P40/2 (hydrogen sulfide, chlorine, and fluoride), P30/2 (alcohol, combustion products, aldehydes, and hydrogen sulfide), T40/2 (chloride and fluoride, fluoride), T40/1 (fluoride), and TA/2 (alcohols) [37]. Each sensor was designed to detect specific analytes, ranging from alcohols and amines to sulfides and fluorides, providing a comprehensive monitoring solution. For each chicken soup sample, quintuplicate analyses were conducted. The measured data were extracted and subjected to principal component analysis (PCA) using the Alpha Soft statistical software (Alpha M.O.S., Version 2012.45). Statistical significance was assessed through Duncan’s multiple range test, with significance established at *p* < 0.05.

#### 3.4.2. E-Tongue Analysis

The Astree E-tongue featured a distinct set of seven sensors (AHS-sources, TS-Salsalami, NMS-umami, PKS, CPS, ANS, and SCS) along with Ag/AgCl serving as the reference electrode. These sensors are specifically designed to identify acidity, saltiness, and freshness. To prepare each 10.0 g chicken soup sample, it was immersed in 100 mL of deionized water in a 100 mL beaker. Following 30 min of ultrasonic extraction, the filtered supernatant was prepared for subsequent analysis. A volume of 80 mL of the filtrate was placed in a special beaker on an E-tongue for analysis. Each sample underwent eight measurements, and the final five stable values were considered as the test results. The response intensities of the E-tongue sensors were normalized to obtain the relative influence intensity.

#### 3.4.3. Free Amino Acid (FAA) Compositional Analysis

The detection of FAA followed a modified version of the procedure outlined in [30]. For each 1.0 g of a chicken soup sample, a 50-mL centrifuge tube received 40 mL of 0.01 mol/L HCl, which was mixed for 5 min. After centrifugation at 10,000 rpm for 4 min, the supernatant was collected, followed by dilution with deionized water in a 10-mL volumetric flask. A 1-mL aliquot of the diluted supernatant was filtered through a 0.22-μm Millipore filter (EMD Millipore, Billerica, MA, USA) for FAA determination.

#### 3.4.4. GC–IMS

Each 2.0 g of a chicken soup sample was weighed, placed in a 20-mL headspace vial, and heated to 60 °C for 15 min. Then, 500 μL of headspace gas was drawn and injected into the sensor array. The analysis of each sample occurred in a column (Fs-SE-54-CB-1; 15 m × 0.53 mm) at 60 °C. The carried gas velocity began at 2.0 mL/min for 2 min, increased to 10.0 mL/min over the next 10 min, and further escalated to 100.0 mL/min from 10 to 40 min. High-purity nitrogen (>99.999%) served as the drift gas at a flow rate of 150 mL/min in the drift tube, which measured 9.8 cm in length and was maintained at a linear voltage of 500 V/cm. Both the IMS detector and drift tube temperature were set at 45 °C.

#### 3.4.5. Identification of Key Volatile Aroma Compounds

The relative odds activity value (ROAV) method was employed to identify key aroma compounds in chicken soup at different stewing temperatures. The ROAV of aroma compounds contributing significantly to chicken soup was established as 100, and the ROAV for other aroma compounds was computed using the following formula [30]:

$$ROAV=\frac{100\times T_{stan}\times C_{m}}{C_{stan}\times T_{m}}$$

where C_stan_ is the relative content of aroma compounds in the sample (%); T_stan_ is the odor threshold in water (μg/kg), as documented in the literature [37]; C_m_ is the highest ROAV among the aroma compounds that contributes significantly to the overall flavor of the chicken soup; T_m_ is the odor threshold of the aroma compound with the most substantial contribution to the overall flavor of the chicken soup sample. The ROAV scale ranges from 0 to 100, with a higher ROAV value suggesting a more significant impact on the overall taste profile of the chicken soup sample. Aroma compounds with ROAV ≥ 1 are considered key volatile aroma components.

### 3.5. Statistical Analysis

The experimental findings are expressed as mean ± standard deviation (SD) using IBM SPSS software (version 20.0, IBM Corp., Armonk, NY, USA). Data from the samples, measured using an E-nose and E-tongue, underwent PCA directly using the instrument’s software. Mapping was performed using the Origin 2021 software. The choice of PCA for our chemometric analysis was based on its simplicity and interpretability in extracting data patterns [38], emphasizing aroma similarities between Ceramic Pot and Combi Oven chicken soup samples. While Partial Least Squares Discriminant Analysis (PLS-DA) and Linear Discriminant Analysis (LDA) are recognized for their stronger discrimination capabilities [39], considering the limited sample size and experimental setup, PCA was deemed sufficient to achieve our objectives and yield insightful results. The chicken soup underwent qualitative analysis while utilizing the Laboratory Analytical Viewer software (VOCal 0.2.9) integrated with GC-IMS NIST/IMS databases. Correlations among E-nose and E-tongue sensors, as well as key aroma and taste substances with a taste active value (TAV) ≥ 1 and ROAV ≥ 1, were explored through partial least squares regression (PLSR). PLSR analysis was performed using the Unscrambler X software (version 10.4, available at https://the-unscrambler-x.software.informer.com/10.4/, accessed on 21 April 2020).

## 4. Conclusions

In this study, chicken soup was cooked via CP, EPC, and EC. E-nose and GC–IMS were used to detect volatile aroma compounds. A total of 39 volatile aroma compounds were detected, among which 23 key aroma compounds were identified. An automatic amino acid analyzer was used to identify FAAs in Sample CP-3 that had significant contributions to the overall taste. Cluster analysis showed that the aroma compounds and FAA of the CP and CO samples were relatively similar. Pearson’s correlation analyses revealed distinct correlations between various amino acids (e.g., glutamic acid, serine, and glycine) and specific volatile compounds. This study provides data for the one-touch cooking of chicken soup in a versatile steam oven. Future studies should investigate the impact of diverse ingredients and recipe variations in the flavor profiles of chicken soup prepared using combi ovens. Comparative analyses of cooking methods contribute to optimal cooking techniques. Further research should focus on sensory evaluation and consumer acceptance to guide culinary practices and enhance satisfaction.

## Figures and Tables

**Table 1:** Effects of different cooking methods on free acid content and TAV values in chicken soup: TAV Taste Active Value, CP, ceramic pot; EPC, electric pressure cooker; CO, combi oven.

Taste	Name	Threshold Value mg/10 g	Free Amino Acid Content (mg/10 g)								TAV									
			CP-1	CP-2	CP-3	EPC-1	EPC-2	EPC-3	CO-1	CO-2	CO-3	CP-1	CP-2	CP-3	EPC-1	EPC-2	EPC-3	CO-1	CO-2	CO-3
umami	Asp	10.00	8.68 ± 0.32 ^a^	8.99 ± 0.32 ^a^	9.87 ± 0.42 ^a^	6.81 ± 0.28 ^b^	7.20 ± 0.31 ^b^	7.14 ± 0.28 ^b^	7.34 ± 0.23 ^b^	7.66 ± 0.34 ^b^	9.42 ± 0.43 ^a^	0.77	0.90	0.99	0.68	0.72	0.71	0.73	0.77	0.94
	Glu	3.00	6.32 ± 0.14 ^c^	7.39 ± 0.36 ^b^	9.22 ± 0.32 ^a^	6.21 ± 0.31 ^c^	5.77 ± 0.23 ^c^	6.03 ± 0.19 ^c^	5.88 ± 0.32 ^c^	7.35 ± 0.25 ^b^	8.82 ± 0.36 ^a^	2.11	2.46	3.07	2.07	1.92	2.01	1.96	2.45	2.94
	Total		14.00	16.38	19.09	13.02	12.97	13.17	13.22	15.01	18.24									
Sweet	Thr	26.00	1.23 ± 0.07 ^c^	2.10 ± 0.11 ^a^	2.04 ± 0.06 ^a^	1.48 ± 0.08 ^b^	1.53 ± 0.05 ^b^	1.63 ± 0.12 ^b^	1.61 ± 0.09 ^b^	1.73 ± 0.12 ^b^	2.12 ± 0.10 ^a^	0.05	0.08	0.08	0.06	0.06	0.06	0.06	0.07	0.08
	Ser	15.00	0.92 ± 0.06 ^a^	0.81 ± 0.05 ^a^	0.86 ± 0.11 ^a^	0.53 ± 0.03 ^b^	0.59 ± 0.06 ^b^	0.60 ± 0.04 ^b^	0.68 ± 0.03 ^b^	0.72 ± 0.03 ^b^	0.86 ± 0.04 ^a^	0.06	0.05	0.06	0.04	0.04	0.04	0.05	0.05	0.06
	Gly	13.00	3.12 ± 0.15 ^a^	3.29 ± 0.13 ^a^	3.29 ± 0.14 a	2.60 ± 0.12 ^b^	2.39 ± 0.17 ^b^	0	2.59 ± 0.21 ^b^	2.72 ± 0.14 ^b^	3.21 ± 0.12 ^a^	0.24	0.25	0.25	0.20	0.18	--	0.20	0.21	0.25
	Ala	6.00	5.55 ± 0.23 ^a^	5.42 ± 0.21 ^a^	5.50 ± 0.19 ^a^	4.41 ± 0.22 ^b^	3.78 ± 0.17 ^c^	3.90 ± 0.16 ^c^	3.90 ± 0.17 ^c^	4.18 ± 0.35 ^b^	5.40 ± 0.21 ^a^	0.92	0.90	0.92	0.76	0.63	0.65	0.65	0.70	0.9
	Total		10.82	11.62	11.69	9.02	8.29	6.13	8.78	9.35	11.58									
bitter	Val	4.00	0	0	1.81 ± 0.09 ^a^	0	0	1.16 ± 0.06 ^b^	1.08 ± 0.04 ^b^	1.23 ± 0.06 ^b^	1.64 ± 0.08 ^a^	0	0	0.45	0	0	0.29	0.27	0.31	0.41
	Ile	9.00	1.36 ± 0.06 ^a^	1.33 ± 0.06 ^a^	1.29 ± 0.05 ^a^	0.71 ± 0.03 ^b^	0.81 ± 0.05 ^b^	0.94 ± 0.03 ^b^	0.91 ± 0.07 ^b^	0.91 ± 0.05 ^b^	1.18 ± 0.06 ^a^	0.15	0.15	0.14	0.08	0.09	0.10	0.10	0.10	0.13
	Leu	19.00	2.28 ± 0.14 ^a^	2.45 ± 0.12 ^a^	2.39 ± 0.13 ^a^	0	1.46 ± 0.07 ^b^	1.61 ± 0.07 ^b^	1.80 ± 0.13 ^b^	1.59 ± 0.08 ^b^	2.05 ± 0.09 ^a^	0.12	0.13	0.13	0	0.08	0.08	0.09	0.08	0.11
	Tyr	26.00	1.35 ± 0.08 ^a^	1.43 ± 0.08 ^a^	1.49 ± 0.06 ^a^	0.93 ± 0.04 ^b^	1.00 ± 0.06 ^b^	1.06 ± 0.05 ^b^	1.06 ± 0.05 ^b^	1.05 ± 0.04 ^b^	1.50 ± 0.06 ^a^	0.05	0.06	0	0.04	0.04	0.04	0.04	0.04	0.0
	Phe	9.00	1.05 ± 0.06 ^a^	1.18 ± 0.11 ^a^	1.22 ± 0.09 ^a^	0.78 ± 0.03 ^b^	0.90 ± 0.03 ^b^	0.83 ± 0.03 ^b^	0.81 ± 0.03 ^b^	0.91 ± 0.05 ^b^	1.34 ± 0.05 ^a^	0.12	0.13	0.14	0.09	0.10	0.09	0.09	0.10	0.15
	His	2.00	1.17 ± 0.04 ^a^	1.26 ± 0.02 ^a^	1.44 ± 0.07 ^a^	0.95 ± 0.06 ^b^	1.14 ± 0.05 ^a^	1.19 ± 0.11 ^a^	1.03 ± 0.06 ^b^	0.93 ± 0.08 ^b^	1.29 ± 0.06 ^a^	0.59	0.63	0.72	0.48	0.57	0.60	0.52	0.47	0.65
	Lys	5.00	0.00	4.69 ± 0.23 ^a^	0.30 ± 0.04 ^e^	0.13 ± 0.02 ^f^	2.30 ± 0.13 ^b^	0.92 ± 0.04 ^c^	0.16 ± 0.02 ^f^	0.61 ± 0.02 ^d^	0.11 ± 0.02 ^f^	0.00	0.94	0.06	0.03	0.46	0.18	0.03	0.12	0.02
	Arg	5.00	1.36 ± 0.12 ^d^	1.72 ± 0.08 ^c^	2.03 ± 0.13 ^b^	3.02 ± 0.19 ^a^	2.05 ± 0.11 ^b^	1.16 ± 0.13 ^e^	1.52 ± 0.11 ^c^	0.00	2.15 ± 0.13 ^b^	0.27	0.34	0.41	0.60	0.41	0.23	0.30	0	0.43
	Total		8.57	14.06	11.97	6.52	9.66	8.87	8.37	7.23	11.26									
Free amino acid content (TFAA)			33.39	42.06	42.75	28.56	30.92	28.17	30.37	31.59	41.08									

Note: CP1-3 represent Sample CP1-3 cooked for 2–4 h with a ceramic pot, respectively; EPC1-3 represent Sample EPC1-3, cooked for 2–4 h with an electric pressure cooker, respectively; CO1-3 represent Sample CO1-3, cooked for 2–4 h with a combi oven, respectively; Different letters (a–f) indicate significant differences in the content of free acid.

**Table 2:** Parameters of the cooking methods for chicken soup.

Number	Equipment	Stewing Time/h	Sample Number
1	Ceramic pot (CP)	2	CP-1
2		3	CP-2
3		4	CP-3
4	Electric pressure cooker (EPC)	2	EPC-1
5		3	EPC-2
6		4	EPC-3
7	Combi oven (CO)	2	CO-1
8		3	CO-2
9		4	CO-3

Note: CP1-3 represent Sample CP1-3, cooked for 2–4 h with a ceramic pot, respectively; EPC1-3 represent Sample EPC1-3, cooked for 2–4 h with an electric pressure cooker, respectively; CO1-3 represent Sample CO1-3, cooked for 2–4 h with combi oven, respectively.

## Data Availability

Data are contained within the article and Supplementary Materials.

## Supplementary Materials

The following supporting information can be downloaded at: https://www.mdpi.com/article/10.3390/molecules29071532/s1, Table S1. Volatile compounds detected by gas phase ion migration spectrum of the chicken soup.
