# Supplementary material for: Effect of Different Cooking Methods on the Aroma and Taste of Chicken Broth
Source: Molecules. 2024 Mar 29;29(7):1532. doi: 10.3390/molecules29071532 (PMC11013132; doi:10.3390/molecules29071532)
Supplement: Supplementary file 1 [file molecules-29-01532-s001.zip › molecules-2940237-supplementary.pdf]

molecules

MDPI

Table S1. Volatile compounds detected by gas phase ion migration spectrum of the chicken soup.

	Compound	CAS#	Formula	RI[a]	Rt [sec][b]	Dt [a.u.][c]
**aldehydes**	(E)-2-octenal	C2548870	$C_8H_{14}O$	1063.5	623.887	1.33467
	(E)-2-heptenal-M	C18829555	$C_7H_{12}O$	959.1	431.283	1.26245
	(E)-2-heptenal-D	C18829555	$C_7H_{12}O$	958.4	430.231	1.66832
	octanal	C124130	$C_8H_{16}O$	1009.2	509.167	1.8142
	heptanal	C111717	$C_7H_{14}O$	904.3	356.557	1.33611
	hexanal	C66251	$C_6H_{12}O$	800.7	260.782	1.5571
	phenylacetaldehyde	C122781	C_8H_8O	1033.8	561.06	1.264
	(E)-2-hexenal	C6728263	$C_6H_{10}O$	848	301.723	1.18118
	benzaldehyde	C100527	C_7H_6O	960.3	432.924	1.15027
	3-methylthiopropanal	C3268493	C_4H_8OS	902.8	354.449	1.40313
	Pentanal	C110623	$C_5H_{10}O$	708.1	195.659	1.19996
	(E)-2-pentenal	C1576870	C_5H_8O	749.2	223.151	1.11079
	nonanal	C124196	$C_9H_{18}O$	1110.7	723.767	1.93084
	3-methylbutanal	C590863	$C_5H_{10}O$	654.1	170.203	1.18547
**ketone**	2,3-butanedione	C431038	$C_4H_6O_2$	580.3	143.547	1.19333
	heptan-2-one	C110430	$C_7H_{14}O$	902.3	353.836	1.61955
	butan-2-one	C78933	C_4H_8O	594.6	148.721	1.24204
	4-hydroxy-2,5-dimethyl-3(2 H)-furanone	C3658773	$C_6H_8O_3$	1069.3	636.219	1.19185
	2-heptanone	C110430	$C_7H_{14}O$	890.8	338.792	1.26018
	2-Butanone	C78933	C_4H_8O	599.6	150.533	1.05537
	butane-2,3-d ione	C431038	$C_4H_6O_2$	593.9	148.463	1.153
**alcohols**	oct-1-en-3-ol	C3391864	$C_8H_{16}O$	989.2	472.33	1.15701
	2-Octanol	C123966	$C_8H_{18}O$	993.6	478.293	1.43073
	pentan-1-ol	C71410	$C_5H_{12}O$	765	233.671	1.25517
	2-Propanol	C67630	C_3H_8O	527.1	124.333	1.09575
	3-Methyl-3-buten-1-ol	C763326	$C_5H_{10}O$	730.4	210.567	1.24437
	Linalool oxide	C60047178	$C_{10}H_{18}O_2$	1063.7	624.451	1.8143
	2-Furanmethanol, 5-methyl-	C3857258	$C_6H_8O_2$	958.6	430.576	1.56886
	Linalool oxide	C60047178	$C_{10}H_{18}O_2$	1063.7	624.451	1.8143
	1.8-Cineole	C470826	$C_{10}H_{18}O$	1030.2	553.572	1.29939
**Acids**	Propanoic acid	C79094	$C_3H_6O_2$	698.7	189.416	1.26221
	2-methylbutanoic acid	C116530	$C_5H_{10}O_2$	831.8	287.69	1.46754
**Heterocyclic**	2-Pentylfuran	C3777693	$C_9H_{14}O$	996.8	482.905	1.25403
	2-acetylthiazol	C24295032	C_5H_5NOS	1008.3	507.112	1.48095
**ethers**	dipropyl disulfide	C629196	$C_6H_{14}S_2$	1113.2	729.135	1.48488
	tert-butylmethylether	C1634044	$C_5H_{12}O$	555.4	134.555	1.13285
**others**	Octamethylcyclotetrasilox ane	C556672	$C_8H_{24}O_4Si_4$	1013.2	517.449	1.67822
	acetic acid ethyl ester	C141786	$C_4H_8O_2$	608.2	153.625	1.0974
	Dimethylformamide	C68122	C_3H_7NO	788	249.83	1.26265

[a]RI : retention index on polar ZB-Wax column; [b]Rt : retention time in capillary GC column; [c]Dt: drift time in drift tube.
